# Association between different proportions of crescents and adverse renal outcomes in immunoglobulin a nephropathy: a systematic review and meta-analysis

**DOI:** 10.1080/0886022X.2025.2495104

**Published:** 2025-05-21

**Authors:** Zhiyun Zang, Kewei Fang, Niya Ma, Yunyun Zhang, Ying Shu, Zi Li

**Affiliations:** a Department of Nephrology, The Third People’s Hospital of Chengdu, Chengdu, Sichuan, China; b Department of Radiology, West China School of Public Health and West China Fourth Hospital, Sichuan University, Chengdu, China; c Department of Nephrology, Institute of Nephrology, West China Hospital of Sichuan University, Chengdu, China

**Keywords:** Immunoglobulin A nephropathy, crescents, renal outcomes, systematic review, meta-analysis

## Abstract

**Objectives:**

Immunoglobulin A nephropathy (IgAN) had several pathological predictive factors, but the predictive value of crescents (C) for renal prognosis was controversial. We conducted a systematic review and meta-analysis to identify the association between crescents and renal outcomes of IgAN patients.

**Methods:**

We searched PubMed, the Cochrane library and Ovid from database inception through to August 30, 2024. The analysis utilized risk ratio (RR) or mean difference (MD) along with the corresponding 95% confidence interval (CI). Data analysis was performed using Stata18 MP (Stata Corp) and RevMan 5.4. The proportion of crescents was classified as C0 (no crescents), C1 (crescents <25%), and C2 (crescents ≥25%).

**Results:**

Eighteen studies were eligible, including 7,567 patients in the C0 group and 4,219 patients in the C group. Our meta-analyses revealed that compared to the C group, the C0 group had significantly lower incidence of composite kidney endpoint [RR = 0.56, 95% CI (0.45, 0.70)] and end-stage renal disease (ESRD) [RR = 0.64, 95% CI (0.51, 0.80)]. The C0 group had significantly higher estimated glomerular filtration rate (eGFR), lower proteinuria, and milder E1, S1, and T1/2 (all *P* values < 0.05). Compared to the C0 or C1 group, the C2 group was more likely to have composite kidney endpoint and ESRD. We did not find the benefit of immunosuppressant therapy (IST) in the C1, C2, or C group.

**Conclusions:**

We found that IgAN patients with crescents had higher risk of composite kidney endpoints and ESRD, especially patients with C2 lesions. IST may not improve renal outcomes in IgAN patients with C1 or C2 lesions.

## Introduction

Immunoglobulin A nephropathy (IgAN) is one of the most prevalent primary glomerulonephritis worldwide and most patients (24–41%) progressed to kidney failure within 10–15 years [1–4]. IgAN is a very heterogeneous disease with complex clinical manifestations, different histological patterns and prognosis. For clinician, it is very important to identify IgAN patients at risk of progression and adopt appropriate therapeutic strategy [3]. The histological features at renal biopsy have shown significant value in risk stratification for IgAN patients [5]. The 2009 Oxford classification identified mesangial hypercellularity (M), endocapillary proliferation (E), segmental glomerulosclerosis (S), and tubular atrophy/interstitial fibrosis (T) as independent prognostic markers [6]. However, the predictive value of crescents (C) for renal prognosis in IgAN was not determined.

Crescents can be observed in approximately 60% of biopsies from IgAN patients [7,8], which have been addressed as a predictor of poor kidney survival in some articles [7,9]. Therefore, crescent was included in the updated Oxford classification in 2017, and the risk stratification strategy was refined into MEST-C score [10]. The proportion of crescents was classified as follows: C0 (no crescents); C1 (crescents in less than one-fourth of glomeruli); and C2 (crescents in one-fourth or more of glomeruli) [8]. However, the predictive value of crescents in IgAN is still controversial, whether for the renal prognosis or treatment guidance. Even in studies that have found a significant association between crescents and prognosis [7,9], it has not been demonstrated whether crescent was a decisive factor for predicting prognosis. Furthermore, several studies have failed to replicate the association between crescents and adverse renal prognosis in IgAN patients [11,12]. Additionally, Kidney Disease: Improving Global Outcomes (KDIGO) guideline suggests that there is insufficient evidence to make treatment decisions based on the presence and score of crescents in the kidney biopsy [5]. But Peng et al. found that immunosuppressant therapy (IST) was associated with a higher remission rate in patients with crescents [13]. Park et al. demonstrated that the association of crescents and renal outcome was better observed in IgAN patients who received IST [14]. Nevertheless, the extent of crescents contributing to treatment decisions remains unclear.

Herein, the present study aimed to perform a systematic review and meta-analysis to explore the association between crescents and renal outcomes for IgAN patients.

## Methods

### Search strategy

We performed a systematic review and meta-analysis of the literatures according to the Preferred Reporting Items for Systematic Reviews and Meta-Analyses (PRISMA) statement [15]. Our meta-analysis has been registered on PROSPERO (CRD42024599209). Eligible studies related to IgAN and crescents were identified by searching PubMed, the Cochrane library and Ovid from database inception through to August 30, 2024 using medical subject headings and text words. For studies that were difficult to obtain the full text, we requested it by sending emails to the corresponding authors.

Search terms included: IgA Nephropathy; Glomerulonephritides, IGA; Berger Disease; Berger’s Disease; Bergers Disease; IGA Glomerulonephritis; IGA Nephropathy; Iga Nephropathy 1; Nephropathy 1, Iga; Immunoglobulin A Nephropathy; Nephropathy, Immunoglobulin A; Nephritis, IGA Type; IGA Type Nephritis; Nephropathy, IGA; Crescent; Extracapillary proliferation; Extracapillary hypercellularity.

### Inclusion and exclusion criteria

Studies eligible for this meta-analysis were those: 1) enrolling participants with biopsy-proven IgAN; 2) comparing the clinical outcomes of different proportions of crescents; 3) providing data on any of the prespecified primary and secondary outcomes.

Studies that did not investigate crescents as a variable were excluded. What is more, studies meeting the following criteria were excluded: duplication, basic research studies, non-English studies, pediatric studies, case reports, meta-analysis, reviews and editorials.

### Data extraction and quality assessment

The literature search, data extraction, and quality assessment were performed by two authors (Zhiyun Zang and Kewei Fang) independently. The extracted data included first author, published year, country or area, the proportion of C0/C1/C2, and general demographic characteristics in each group, using a standardized data form. Any disagreements in abstract screening and data extraction were resolved by another author (Zi Li).

We adopted the Newcastle-Ottawa Scale (NOS) to assess the methodological quality of the included studies [16]. It comprised the study of choice (four items, four points), group comparability (one item, two points), and outcome measurements (three items, three points). The maximum of 9 points represented the highest quality of methodology. The NOS score of 7 or more indicated that the quality of methodology was good. The score between 4 and 7 indicated that the quality of methodology was moderate. A score less than 4 indicated poor methodological quality.

### Outcomes

The primary outcomes were composite kidney endpoint and end-stage renal disease (ESRD) in patients with different C scores. The secondary outcomes were composite kidney endpoint and ESRD in patients using IST. The composite kidney endpoint included the development of ESRD, ≥ 50% decrease in estimated glomerular filtration rate (eGFR), doubling of serum creatinine, renal replacement therapy initiation or death. IST was defined as the use of any immunosuppressive agent, which included glucocorticoid (GC), cyclophosphamide (CTX), mycophenolate mofetil (MMF), cyclosporin A (CsA), tacrolimus (FK506), azathioprine (AZA), etc. In addition, we analyzed the eGFR, proteinuria, and pathological characteristics of patients with different proportions of crescents.

### Statistical analysis

For studies that only provide median values, the estimations of the mean and standard deviation were according to Wan et al.’s method [17] and the formulas were provided in Supplementary Table 1. The potential heterogeneity of the studies was assessed by *I*^2^ statistics which was used to quantify between-study heterogeneity. *I*^2^ values of 50% or less and greater than 50% were considered as having low and high heterogeneity, respectively. Fixed-effects models were used for low heterogeneity, and the random effect models were used for high heterogeneity. The network meta-analysis was conducted to compare the renal outcomes in C0, C1 and C2 groups together. Moreover, between-study heterogeneity was further explored using subgroup analysis and leave-one-out meta-analysis. Publication bias was evaluated using funnel plot analysis and Egger’s test. For the results with significant publication bias, we further conducted the trim-and-fill analysis. In this study, we reported results as risk ratio (RR) or mean difference (MD) and their 95% confidence intervals (CIs). Data analysis was performed using Stata18 MP (Stata Corp) and RevMan 5.4. Statistical significance was set at *p* < 0.05, and all tests were two-tailed.

## Results

### Search results

**Figure 1.**
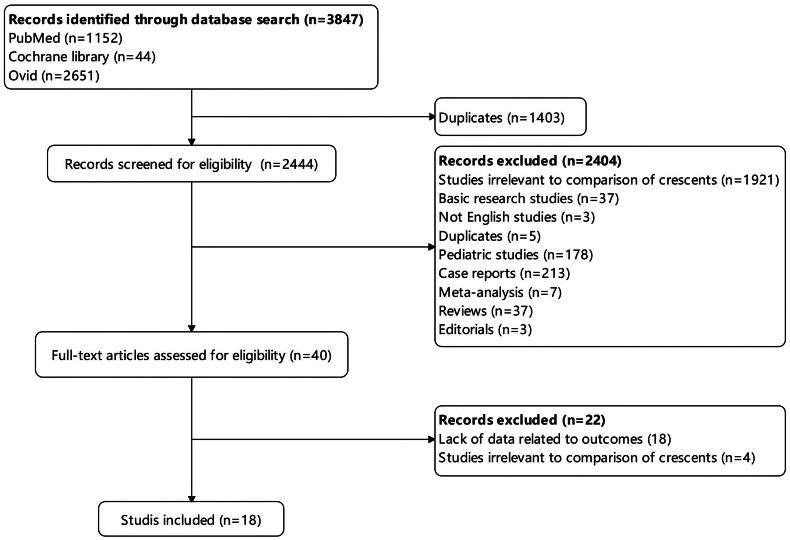
Literature search and study selection.

As shown in Figure 1, 3,847 articles were identified after initial search, and 1,403 articles were duplicates. Of the remaining 2,444 articles, 2,404 of them were excluded after screening the titles and abstracts. Eventually, 18 eligible researches were included [12–14,18–32] after detailed evaluation. The study from Schimpf [18] was a secondary analysis of randomized STOP-IgAN trial. The remaining articles about crescents in IgAN patients were retrospective studies. There were 4 multicenter and 14 single-center studies. The studies were conducted in countries in Asia, Europe, North America and South America.

### Characteristics of included studies

11,786 IgAN patients were enrolled in the study, including 7,567 patients in the C0 group and 4,219 patients in the C group. The proportion of patients with crescents was 35.8%. The sample size of enrolled studies ranged from 56 to 3,380 patients, with 5 research papers having more than 1,000 patients enrolled. In term of demographics, the percentage of males was 49.1% and the average age was 34.7 years old. The characteristics of the eligible studies are shown in Table 1.

**Table 1.** Characteristics of the eligible studies.

Author/Year	Study design	Study period	Ethnicity	Total	Length of follow-up	Clinical outcomes	C0	C1	C2	C	NOS
Bitencourt-Dias et al. 2004 [32]	Single-centre	1980–2001	North American	56	NA	②	30	NA	NA	26	7
Chen et al. 2020 [24]	Single-centre	2003–2013	Asian	388	7.2 ± 3.1 years	②④	343	35	10	45	8
Chen et al. 2022 [31]	Single-centre	2017–2019	Asian	144	NA	①②	59	77	8	85	8
Du et al. 2022 [28]	Single-centre	2007–2019	Asian	1242	43 months (range 6–151)	①②	647	493	102	595	8
Guo et al. 2023 [19]	Single-centre	1994–2016	Asian	1262	3.8 years	①②	518	596	148	744	8
Lee et al. 2014 [30]	Single-centre	2000–2009	Asian	430	61.0 ± 32.3 months	①②	349	NA	NA	81	8
Lim et al. 2020 [26]	Multicenter	2011–2016	Southeast Asian	145	27 (12, 46) months	①②	101	38	6	44	7
Ma et al. 2020 [23]	Single-centre	2008–2013	Asian	338	49.9 ± 26.0 months	①②	169	142	27	169	8
Neves et al. 2020 [22]	Single-centre	1996–2016	North American	111	64 (20-106) months	②③	80	27	4	31	8
Parka et al. 2019 [14]	Multicenter	NA	Asian	3380	8.74 (5.44–12.24) years	①②	2656	664	60	724	7
Peng et al. 2019 [13]	Single-centre	2008–2015	Asian	1328	46.1 ± 23.6 months	①②	993	257	78	335	8
Ramani et al. 2022 [20]	Single-centre	1992–2020	North American	73	8.4 ± 7 years	②	47	21	5	26	8
Ruan et al. 2022 [21]	Single-centre	2010–2021	Asian	458	54.7 ± 20.4 months	①②	255	187	16	203	7
Schimpf et al. 2018 [18]	Multicenter	2008–2011	European	70	NA	②	48	17	5	22	7
Ştefan et al. [25]	Multicenter	2003–2013	European	121	59.7 (51.8–67.6) months	③⑤	83	NA	NA	38	8
Wang et al. 2021 [27]	Single-centre	2004–2019	Asian	100	29.5 months	②	16	24	60	84	8
Zhang et al. 2017 [29]	Single-centre	2000–2011	Asian	988	49 months (range 4–96)	②③④	554	NA	NA	434	8
Zhang et al. 2018 [12]	Single-centre	1989–2014	Asian	1152	45 (25–70) months	①②	619	447	86	533	8

Clinical outcomes: ① ≥ 50%decrease in the eGFR; ② ESRD; ③ Doubling of serum creatinine; ④ Death; ⑤ Renal replacement therapy. NA: no data.

Evaluating by NOS criteria, the enrolled studies scored a median of 8 points, which indicated medium quality (Table 1).

#### Predictive value of crescents in renal outcomes of IgAN patients

Table 2 shows the renal outcomes in different cohorts. There were 18 studies comparing the kidney endpoints between the C0 and C groups, and 7 studies comparing the clinical outcomes with different C scores. Furthermore, eGFR, proteinuria and pathological characteristics of patients with different proportions of crescents were provided in Supplementary Table 2 and Supplementary Table 3.

**Table 2.** The clinical outcomes in the C0/1/2 and C cohort.

Author/Year	C0			C1			C2			C		
	Total	ESRD	Composite endpoint	Total	ESRD	Composite endpoint	Total	ESRD	Composite endpoint	Total	ESRD	Composite endpoint
Bitencourt-Dias et al. 2004 [32]	30	3	3	NA	NA	NA	NA	NA	NA	26	17	17
Chen et al. 2020 [24]	343	63	71	35	NA	NA	10	NA	NA	45	14	18
Chen et al. 2022 [31]	59	NA	2	77	NA	NA	8	NA	NA	85	NA	15
Du et al. 2022 [28]	647	NA	34	493	NA	45	102	NA	20	595	NA	65
Guo et al. 2023 [19]	518	45	NA	596	51	NA	148	23	NA	744	74	NA
Lee et al. 2014 [30]	349	32	72	NA	NA	NA	NA	NA	NA	81	17	36
Lim et al. 2020 [26]	101	11	NA	38	NA	NA	6	NA	NA	44	3	NA
Ma et al. 2020 [23]	169	6	12	142	NA	NA	27	NA	NA	169	21	29
Neves et al. 2020 [22]	80	NA	17	27	NA	NA	4	NA	NA	31	NA	16
Parka et al. 2019 [14]	2656	302	509	664	96	168	60	19	23	724	115	191
Peng et al. 2019 [13]	993	153	164	257	35	31	78	28	26	335	63	57
Ramani et al. 2022 [20]	47	11	11	21	NA	NA	5	NA	NA	26	5	5
Ruan et al. 2022 [21]	255	NA	28	187	NA	22	16	NA	4	203	NA	26
Schimpf et al. 2018 [18]	48	4	4	17	NA	NA	5	NA	NA	22	4	4
Ştefan et al. 2016 [25]	83	10	19	NA	NA	NA	NA	NA	NA	38	14	15
Wang et al. 2021 [27]	16	0	0	24	2	2	60	27	27	84	29	29
Zhang et al. 2017 [29]	554	40	NA	NA	NA	NA	NA	NA	NA	434	51	NA
Zhang et al. 2018 [12]	619	80	NA	447	46	NA	86	18	NA	533	64	NA

ESRD, end-stage renal disease; NA, no data.

##### Comparison of renal outcomes for patients with versus without crescents

**Figure 2.**
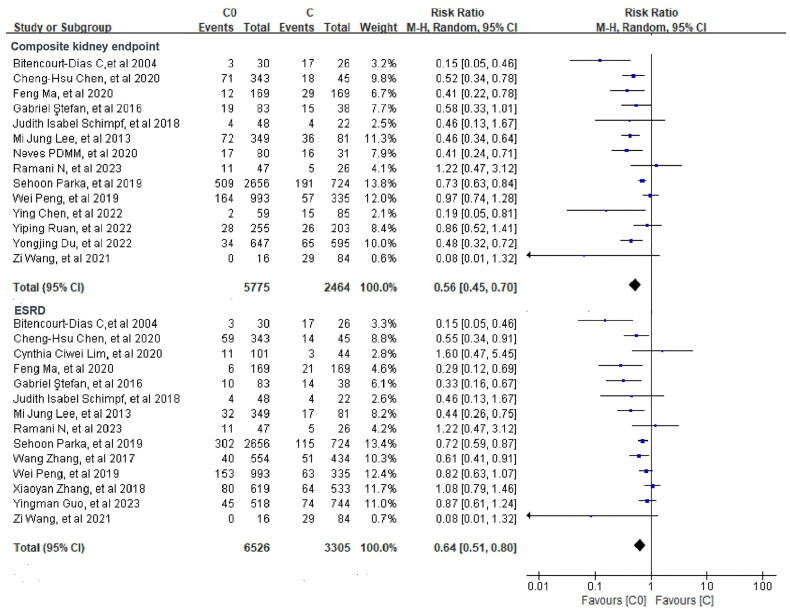
Meta-analyses of the renal outcomes between C0 and C groups.

Our meta-analyses revealed that in comparison to the C group, the C0 group had a significantly lower incidence of composite kidney endpoint [RR = 0.56, 95% CI (0.45, 0.70)] with high heterogeneity (*I*^2^ = 65%). Moreover, we found statistical differences between the two groups in terms of ESRD [RR = 0.64, 95% CI (0.51, 0.80)] and its heterogeneity was also high (*I*^2^ = 64%) (Figure 2).

Furthermore, we found that the C0 group exhibited significantly higher eGFR [MD = 8.21, 95% CI (3.45, 12.96)] and lower proteinuria [MD = −0.30, 95% CI (−0.37, −0.23)]. And the C0 group had milder E1 [RR = 0.36, 95% CI (0.22, 0.57)], S1 [RR = 0.57, 95% CI (0.34, 0.96)], and T1/2 [RR = 0.52, 95% CI (0.37, 0.73)] scores comparing to the C group (Supplementary Fig. 1).

##### Comparison of renal outcomes for patients in C0, C1 and C2 groups

Additionally, we performed network meta-analysis to identify the differences among the groups. The effect sizes for the differences between all groups were presented in league table (Supplementary Table 4). The network meta-analysis suggested that there were no significant differences in composite kidney endpoint, ESRD, eGFR or T1/T2 between C0 and C1 groups. But the patients in C0 group had significantly lower proteinuria [MD = −0.29, 95% CI (−0.39, −0.18)] and pathological scores of M1 [RR = 0.80, 95% CI (0.72, 0.90)], E1 [RR = 0.43, 95% CI (0.22, 0.85)], and S1 [RR = 0.83, 95% CI (0.76, 0.91).

Compared to the C2 group, patients in the C0 group were not inclined to have composite kidney endpoint [RR = 0.40, 95% CI (0.28, 0.57)] and ESRD [RR = 0.45, 95% CI (0.35, 0.57)]. And the C0 group demonstrated significantly higher eGFR [MD = 20.36, 95% CI (14.04, 26.67)] and lower proteinuria [MD = −1.60, 95% CI (−1.84, −1.36)]. What’s more, the C0 group showed milder pathological characteristics in M1 [RR = 0.68, 95% CI (0.60, 0.78)], E1 [RR = 0.28, 95% CI (0.14, 0.56)], and T1/T2 [RR = 0.50, 95% CI (0.39, 0.64)]. There was no significant difference in S1 scores between C0 and C2 groups [RR = 0.92, 95% CI (0.82, 1.03)].

When comparing to C2 group, the patients in the C1 group had lower incidence of composite kidney endpoint [RR = 0.46, 95% CI (0.32, 0.66)] and ESRD [RR = 0.45, 95% CI (0.35, 0.58)]. And the patients in C1 group had higher eGFR [MD = 17.21, 95% CI (10.81, 23.62)], lower proteinuria [MD = −1.31, 95% CI (−1.55, −1.07)], and milder M1 [RR = 0.85, 95% CI (0.75, 0.97)] and T1/2 [RR = 0.61, 95% CI (0.47, 0.81)]. There was no statistical difference in E1 [RR = 0.65, 95% CI (0.33, 1.28)] and S1 [RR = 1.11, 95% CI (0.98, 1.25).

Our network meta-analysis revealed that the C0 group had the highest ranking in all renal outcomes (composite kidney endpoint, ESRD, eGFR, proteinuria and pathological characteristics). And the C2 group had the worst ranking in all renal outcomes except for S1 scores. The patients with C1 lesions had the worst ranking in terms of S1 scores.

#### Renal outcomes of immunosuppressive therapy in IgAN patients with crescents

Only a portion of studies have reported the results of IgAN patients using immunosuppressants. The clinical outcomes of patients in the C0/1/2 cohort with or without immunosuppression were shown in Table 3. The patients in the C2 cohort were more likely to receive immunosuppressive treatment than the C0 or C1 cohort (C0 49.5%, C1 46.7%, and C2 80.6%).

**Table 3.** Clinical outcomes of patients in the C0/1/2 cohort with or without IST.

Clinical outcomes Author/Year		ESRD	Composite endpoint		ESRD	Composite endpoint		ESRD	Composite endpoint		ESRD	Composite endpoint
Guo et al. 2023 [19]	C0 (*n* = 518)			C1 (*n* = 596)			C2 (*n* = 148)			C (*n* = 744)		
	Without IST (*n* = NA)	NA	NA	Without IST (*n* = 387)	18	NA	Without IST(*n* = 36)	2	NA	Without IST (*n* = 423)	20	NA
	IST (*n* = NA)	NA	NA	IST (*n* = 209)	33	NA	IST(*n* = 112)	21	NA	IST (*n* = 321)	54	NA
Lim et al. 2020 [26]	C0 (*n* = 101)			C1 (*n* = NA)			C2 (*n* = NA)			C (*n* = 44)		
	Without IST (*n* = 58)	9	NA	Without IST (*n* = NA)	NA	NA	Without IST (*n* = NA)	NA	NA	Without IST (*n* = 15)	3	NA
	IST (*n* = 43)	2	NA	IST (*n* = NA)	NA	NA	IST (*n* = NA)	NA	NA	IST (*n* = 29)	0	NA
Ma et al. 2020 [23]	C0 (*n* = 169)			C1 (*n* = NA)			C2 (*n* = NA)			C (*n* = 169)		
	Without IST (*n* = 54)	NA	5	Without IST (*n* = NA)	NA	NA	Without IST (*n* = NA)	NA	NA	Without IST (*n* = 36)	NA	6
	IST (*n* = 115)	NA	7	IST (*n* = NA)	NA	NA	IST (*n* = NA)	NA	NA	IST (*n* = 133)	NA	23
Peng et al. 2019 [13]	C0 (*n* = 993)			C1 (*n* = 257)			C2 (*n* = 78)			C (*n* = 335)		
	Without IST (*n* = 456)	82	NA	Without IST (*n* = 59)	10	NA	Without IST (*n* = 9)	5	NA	Without IST (*n* = 68)	15	NA
	IST (*n* = 537)	71	NA	IST (*n* = 198)	25	NA	IST (*n* = 69)	23	NA	IST (*n* = 267)	48	NA
Ruan et al. 2022 [21]	C0 (*n* = 255)			C1 (*n* = 187)			C2 (*n* = 16)			C (*n* = 203)		
	Without IST (*n* = 199)	NA	22	Without IST (*n* = 108)	NA	12	Without IST (*n* = 2)	NA	0	Without IST (*n* = 110)	NA	12
	IST (*n* = 56)	NA	6	IST (*n* = 79)	NA	10	IST (*n* = 14)	NA	4	IST (*n* = 93)	NA	14
Schimpf et al. 2018 [18]	C0 (*n* = 48)			C1 (*n* = NA)			C2 (*n* = NA)			C (*n* = 22)		
	Without IST (*n* = 24)	1	1	Without IST (*n* = NA)	NA	NA	Without IST (*n* = NA)	NA	NA	Without IST (*n* = 8)	3	3
	IST (*n* = 24)	3	3	IST (*n* = NA)	NA	NA	IST (*n* = NA)	NA	NA	IST (*n* = 14)	1	1

We further analyzed the ESRD of patients with and without IST from different crescent cohorts. Our results didn’t find any significance in patients with or without IST in C1, C2, or C group.

**Figure 3.**
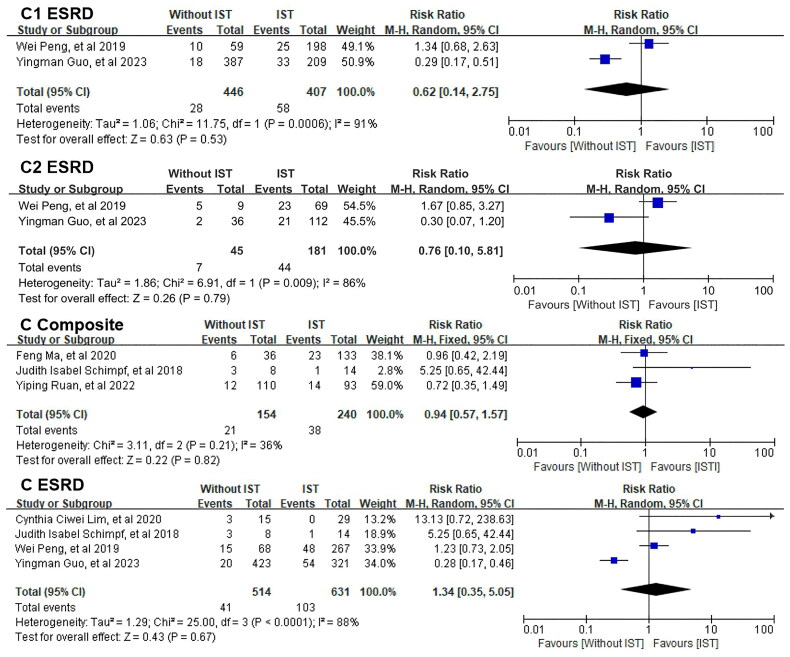
Meta-analyses of the renal outcomes between patients with or without IST.

As for the composite kidney endpoint, there were no differences between patients with or without IST in C group [RR = 0.94, 95% CI (0.57, 1.57), *I*^2^ = 36%] based on data collected from three studies. We did not conduct the meta-analysis about the composite kidney endpoints of C1 or C2 group, because only one study reported relevant data. Figure 3 shows the results.

#### Publication bias

**Figure 4.**
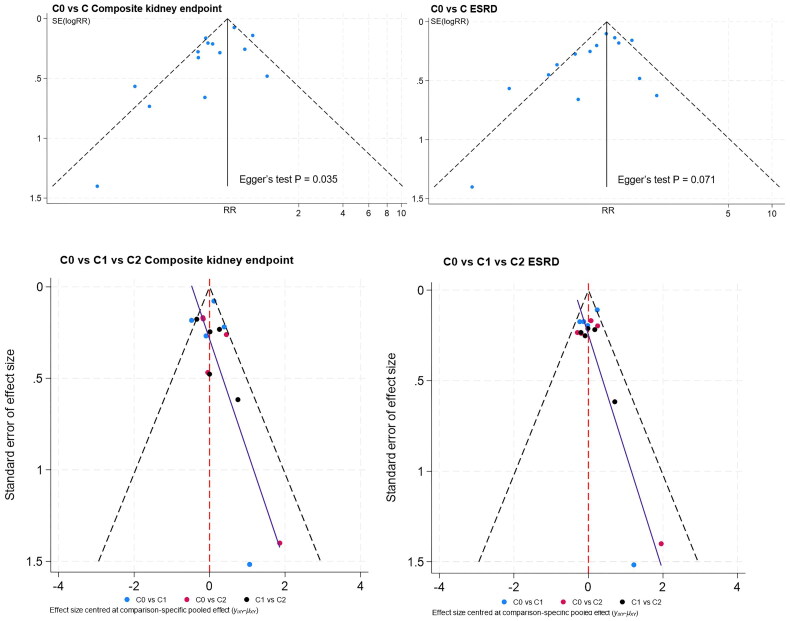
Publication bias of renal outcomes.

**Figure 5.**
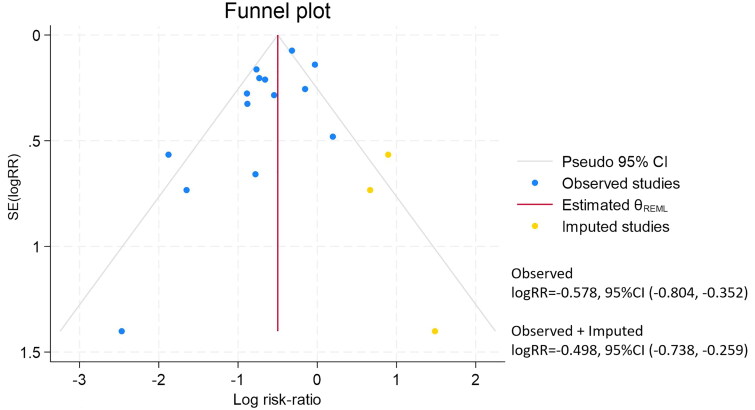
The trim-and-fill analysis of composite kidney endpoint between C0 and C group.

In the comparison of composite kidney endpoint between C0 and C group, the funnel plot was roughly symmetrical. However, the Egger’s test indicated publication bias (*p* = 0.035) (Figure 4). Therefore, we conducted the trim-and-fill analysis. No significant difference was observed after analysis, indicating that publication bias did not have a notable effect on the results (Figure 5). For the remaining primary outcomes, no obvious publication bias was observed in the funnel plots (Figure 4). The funnel plots of other outcomes were roughly symmetrical.

#### Sensitivity analysis

We conducted the leave-one-out meta-analysis to investigate the possible sources of heterogeneity. We didn’t find any study exhibiting significant heterogeneity, which indicated that the results were relatively stable.

**Figure 6.**
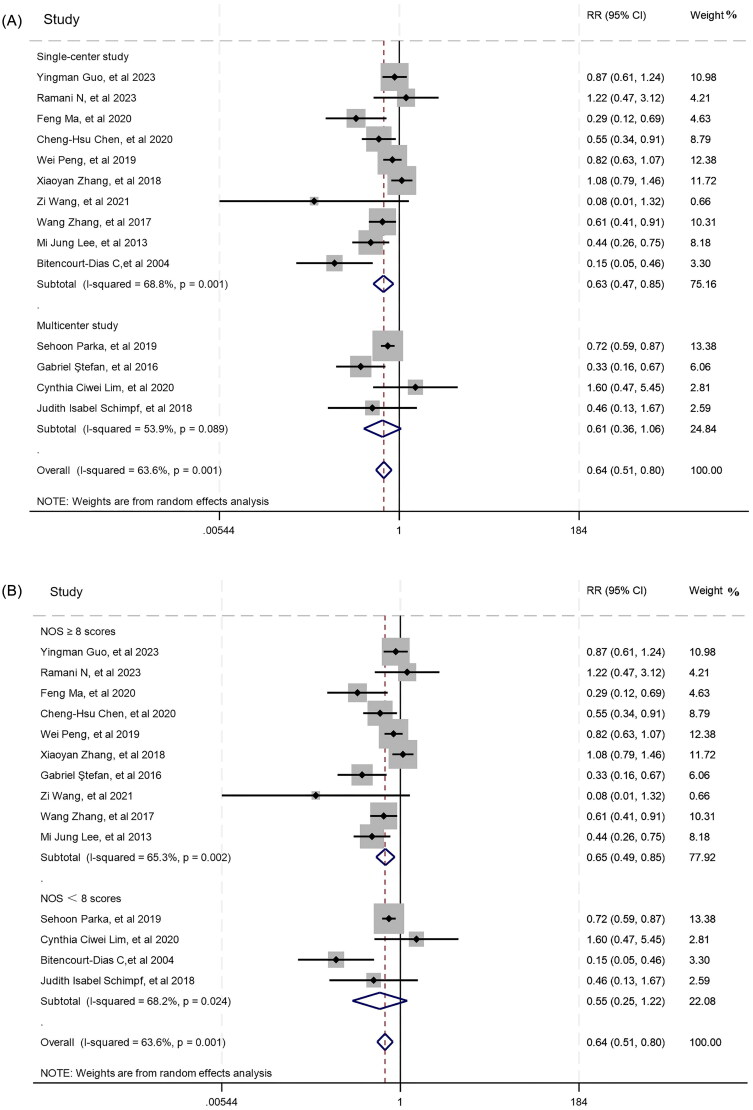
The subgroup analysis of ESRD between C0 and C group: A. Single-center and multicenter studies; B. NOS ≥ 8 and NOS < 8 scores.

Furthermore, we conducted a subgroup analysis for the clinical outcomes between C0 and C groups based on study design (single-center or multicenter study), ethnicity (Asian or non-Asian), and NOS scores (NOS ≥ 8 or NOS < 8 scores). The results showed that these factors had no obvious impact on composite kidney endpoint. Regarding to ESRD, the difference between C and C0 remained significant after dividing into Asian and non-Asian populations. There was no significance between C and C0 patients based on multicenter studies [RR = 0.61, 95% CI (0.36, 1.06), *I*^2^ = 54%] or studies with NOS < 8 scores [RR = 0.55, 95% CI (0.25, 1.22), *I*^2^ = 68%]. More details are shown in Figure 6.

## Discussion

In the present meta-analysis, we found that IgAN patients with crescents had a higher risk of composite kidney endpoints and ESRD, especially patients with C2 scores. Furthermore, we didn’t demonstrate that IST could improve renal outcomes in IgAN patients with C1 or C2 scores.

The previous studies have confirmed that crescents had independent prognostic value for IgAN patients [13,14,33] and our meta-analysis confirmed that crescent formation was closely related to renal prognosis. A possible explanation was that patients with crescents presented with worse clinical characteristics and pathological manifestations. The previous studies reported that C score was significantly correlated with increased proteinuria, decreased eGFR and more severe segmental glomerulosclerosis and tubular atrophy/interstitial fibrosis [21,31], which was consistent with our further analysis. What’s more, we found patients with C lesions had worse endocapillary proliferation. It seems reasonable that patients with crescents have a poorer prognosis because proteinuria, reduced eGFR, and T1/2 are important prognostic factors for IgAN patients [10,34].

In our subgroup analysis, the relationship between crescents and renal prognosis still existed for Asian or non-Asian IgAN populations. Due to the limited enrolled studies and the absence of specific data regarding ethnicity in each study, we were unable to perform a more detailed categorization and analysis of the population. We didn’t observe the association between crescents with ESRD in muticenter studies or studies with NOS < 8 scores. Possible explanations were: 1) The population variations, as multicenter studies may include patients from diverse regions. 2) The studies with NOS < 8 scores may have lower methodological quality and lead to bias. 3) The smaller sample sizes in multicenter studies or studies with NOS < 8 scores resulted in insufficient statistical power. Further high-quality studies from multicenter are required. Additionally, the exclusion criteria between studies were slightly different. Chen et al. did not exclude 6 IgAN patients with positive anti-neutrophil cytoplasmic antibody (ANCA) [24].

Moreover, different proportions of crescents may lead to various prognoses in IgAN patients. Ştefan et al. reported that the presence of crescents, even in small numbers, should be considered as an important renal outcome prognostic factor [25]. This retrospective study confirmed that crescents were independently related to the prognosis of IgAN patients and the optimal proportion of crescents for predicting renal survival was 11%. The proportion of crescents ≥ 11% was an independent risk factor for poor prognosis of IgAN patients [31]. Du et al.’s multivariate Cox regression showed that crescents ≥ 50% was an independent risk factor for ESRD and crescents ≥ 25% was an independent risk factor for the combined renal endpoint (ESRD or ≥ 50% decrease in eGFR) [28]. Zhang et al.’s data showed that crescents with 5% to 9% was still an independent risk of unfavorable prognosis [29]. Our analysis indicated that patients with C2 lesions (crescents ≥ 25%) had the worst kidney outcomes and more severe proteinuria, reduced eGFR, mesangial hypercellularity, endocapillary proliferation, and tubular atrophy/interstitial fibrosis. The results of the present study suggest that patients with C2 lesions should receive more attention. Further exploration of the relationship between the proportion of crescents and renal prognosis is necessary.

Immunosuppressive therapy is crucial in the treatment regimens for IgAN patients, but the presence of crescents does not indicate the commencement of immunosuppression [5]. The previous respective Chinese cohort demonstrated that IgAN patients with C2 lesions had a 1.85 times higher risk of kidney outcomes without IST, while the HR decreased to 0.83 with IST. In addition, IST reduced the risk of kidney outcomes by 70% in patients presented with crescents and fibrinoid necrosis or endocapillary hypercellularity lesions [19]. The STOP-IgAN trial found that ESRD occurred more frequently in supportive therapy group than IST group for C1/C2 patients [18]. Peng et al. also reported that IST was apparently associated with a higher proteinuria remission rate and lower ESRD rate in patients with crescents [13]. However, our meta-analysis didn’t find the benefit of IST in C1/C2 patients, which was inconsistent with the previous studies. On the one hand, the limited eligible studies and samples in the meta-analysis may affect the predictive value of IST in IgAN patients with crescents. Similarly, due to the restricted data, we could not evaluate eGFR, proteinuria, or remission rate after IST treatment in IgAN patients with different C lesions. On the other hand, active glomerular lesions can be reversed by IST in IgAN patients, including crescents [35]. What’s more, calculating the proportions of crescents is not an accurate method which is affected by the biopsy sample size and the number of histologic sections examined [36]. And the strategy of IST varies in different hospitals, which is based on the clinical manifestations, pathological characteristics, and the clinical experience of physicians. The prognostic value of IST in IgAN patients with crescents is still unclear, and further research is needed to determine whether pathological reversal of crescents is beneficial for renal prognosis.

Additionally, our study found that the proportion of patients receiving immunosuppressants in the C0 group (49.5%) was higher than that in the C1 group (46.7%). This observation may be influenced by several factors, including the small sample size and variability in treatment strategies across studies. There were only 6 studies have reported the results of IgAN patients using immunosuppressants, which limited the generalizability and reliability of the findings. What’s more, physicians’ and patients’ preferences for treatment options may contribute to the observed difference between the C0 and C1 groups. The percentages of immunosuppressive therapy use in the C0 group varied across studies (22-68%) [13,18, 21,23,26]. We suggest that future studies should aim to expand the sample size and standardize treatment strategies to ensure more reliable and generalizable results.

There were some limitations in the present study. First, the eligible studies were retrospective and there were few data about crescents in randomized clinical trial settings. Second, the quality of the included studies was variable, but we identified unpublished studies by sending emails to the corresponding authors and conducting the trim-and-fill analysis to reduce the possibility of publication bias. Third, we were unable to perform a subgroup analysis to explore the predictive significance of crescents in different ethnicities and evaluate the importance of IST in crescents sufficiently because of limited studies.

## Conclusions

Crescents were associated with worse composite kidney endpoints and ESRD in IgAN patients, especially C2 lesions. We didn’t find the benefit of IST in patients with C1 or C2 lesions because of limited studies. Further studies are warranted to explore the significance of IST in IgAN patients with crescents.

## Supplementary Material

Supplemental Material

Supplemental Material

Supplemental Material

Supplemental Material

## Data Availability

The data supporting this study’s findings are available from PubMed, the Cochrane Library, and Ovid. Further inquiries can be directed to the corresponding author.
