# Supplementary material for: Association between different proportions of crescents and adverse renal outcomes in immunoglobulin a nephropathy: a systematic review and meta-analysis
Source: Ren Fail. 2025 May 21;47(1):2495104. doi: 10.1080/0886022X.2025.2495104 (PMC12168408; doi:10.1080/0886022X.2025.2495104)

**Supplementary Table 1. The formulas used for estimating the mean and standard deviation**

|  | Scenarios = {a,m,b;n} | Scenarios = {a,q1,m,q3,b;n} | Scenarios = {q1,m,q3;n} |
| --- | --- | --- | --- |
| Mean | $\bar{x}$ ≈ $\frac{a+2m+b}{4}$ | $\bar{x}$ ≈$\frac{a+2q1+2m+2q3+b}{8}$ | $\bar{x}$ ≈ $\frac{q1+m+q3}{3}$ |
| Standard deviation | *s ≈* $\frac{b-a}{2\varphi^{-1}(\frac{n-0.375}{n+0.25})}$ | *s ≈* $\frac{b-a}{4\varphi^{-1}(\frac{n-0.375}{n+0.25})}$+ $\frac{q3-q1}{4\varphi^{-1}(\frac{0.75n-0.125}{n+0.25})}$ | *s ≈* $\frac{q3-q1}{2\varphi^{-1}(\frac{0.75n-0.125}{n+0.25})}$ |

Note: a = the minimum value, q1 = the first quartile, m = the median, q3 = the third quartile, b = the maximum value, n = the sample size.

**Supplementary Table 2. The eGFR and proteinuria of patients with different proportions of crescents**

| **Author/Year** | **C0** | **eGFR** | **Proteinuria** | **C1** | **eGFR** | **Proteinuria** | **C2** | **eGFR** | **Proteinuria** | **C** | **eGFR** | **Proteinuria** |
| --- | --- | --- | --- | --- | --- | --- | --- | --- | --- | --- | --- | --- |
| Bitencourt-Dias, C. et al, 2004 [32] | 30 | NA | 1.2±0.9 | NA | NA | NA | NA | NA | NA | 26 | NA | 4.6 ± 3.4 |
| Chen, CH. et al, 2020 [24] | 343 | 58.05±40.20 | 2.28±2.74 | 35 | NA | NA | 10 | NA | NA | 45 | 50.10±38.15 | 3.74±4.18 |
| Chen, Y. et al, 2022 [31] | 59 | 99.77±33.74 | 0.64±0.93 | 77 | NA | NA | 8 | NA | NA | 85 | 84.59 ± 31.05 | 1.61 ± 2.14 |
| Du, Y. et al, 2022 [28] | 647 | 90±42.4 | 1.38±1.25 | 493 | NA | NA | 102 | NA | NA | 595 | 91.1±48.4 | 1.9±1.6 |
| Guo, Y. et al, 2023 [19] | 518 | 87±29 | 1.2±1.1 | 596 | 83±29 | 1.4±1.2 | 148 | 62±31 | 3±2.3 | 744 | NA | NA |
| Lee, MJ. et al, 2013 [30] | 349 | 82.8 ± 23.4 | 0.9±1.1 | NA | NA | NA | NA | NA | NA | 81 | 70.9 ± 25.2 | 1.6±1.7 |
| Lim, CC. et al, 2020 [26] | 101 | 56.5±35 | 2.2±1.9 | 38 | 57.2±43.9 | 2.9±2.6 | 6 | 46.4±30.8 | 7±8.1 | 44 | 56.6±42.7 | 3.2±3 |
| Ma, F. et al, 2020 [23] | 169 | 85.7±35.2 | 2.1±2.1 | 142 | NA | NA | 27 | NA | NA | 169 | 83.1±30.4 | 2.1±2.9 |
| Neves, PDMM. et al, 2020 [22] | 80 | 62.4±41.7 | 1.9±1.5 | 27 | NA | NA | 4 | NA | NA | 31 | 38±24.8 | 2.6±2.1 |
| Parka, S. et al, 2019 [14] | 2656 | 83.3±37 | 1.2±1.2 | 664 | 82.8±44.1 | 1.6±1.5 | 60 | 54.5±36.6 | 2.7±2.4 | 724 | NA | NA |
| Peng, W. et al, 2019 [13] | 993 | 86.2±34.7 | 2.6±3.0 | 257 | 78.8±32.9 | 3.1±3.1 | 78 | 72.7±40.1 | 4.4±2.9 | 335 | NA | NA |
| Ramani, N. et al, 2023 [20] | 47 | 64±31 | 1.7±1.7 | 21 | NA | NA | 5 | NA | NA | 26 | 61±28 | 2.8±2.7 |
| Ruan, Y. et al, 2022 [21] | 255 | 92.7±32.7 | 0.8±0.9 | 187 | 83.4±33.6 | 1.0±1.1 | 16 | 59.1±31.2 | 2.5±2.3 | 203 | NA | NA |
| Schimpf, JI. et al, 2018 [18] | 48 | 62±29 | NA | 17 | NA | NA | 5 | NA | NA | 22 | 64 ± 25 | NA |
| Ştefan, G. et al, 2016 [25] | 83 | 48.7±6.8 | 1.8±0.7 | NA | NA | NA | NA | NA | NA | 38 | 35.9±21.4 | 2.9±1.4 |
| Wang, Z. et al, 2021 [27] | 16 | NA | 1.7±2.4 | 24 | NA | 2.1±1.7 | 60 | NA | NA | 84 | NA | NA |
| Zhang, W. et al, 2017 [29] | 554 | 100.7±43.9 | 0.6±0.6 | NA | NA | NA | NA | NA | NA | 434 | 87±43.9 | 0.9±0.9 |
| Zhang, X. et al, 2018 [12] | 619 | 76.7±40.9 | 1.4±1.3 | 447 | 79.7±32.9 | 1.7±1.4 | 86 | 68.3±33.8 | 2.7±1.8 | 533 | NA | NA |

eGFR: ml/min/1.73 m2, mean±SD; Proteinuria: g/24 h, mean±SD; NA: no data.

**Supplementary Table 3. The pathological characteristics of patients with different proportions of crescents**

M1: mesangial hypercellularity; E1: endocapillary proliferation; S1: segmental glomerulosclerosis; T1/T2: tubular atrophy/interstitial fibrosis. NA: no data.

| **Author/Year** | **C0** | **M1** | **E1** | **S1** | **T1/T2** | **C1** | **M1** | **E1** | **S1** | **T1/T2** | **C2** | **M1** | **E1** | **S1** | **T1/T2** | **C** | **M1** | **E1** | **S1** | **T1/T2** |
| --- | --- | --- | --- | --- | --- | --- | --- | --- | --- | --- | --- | --- | --- | --- | --- | --- | --- | --- | --- | --- |
| Bitencourt-Dias, C. et al, 2004 [32] | 30 | NA | NA | NA | NA | NA | NA | NA | NA | NA | NA | NA | NA | NA | NA | 26 | NA | NA | NA | NA |
| Chen, CH. et al, 2020 [24] | 343 | 118 | 48 | 21 | 45 | 35 | NA | NA | NA | NA | 10 | NA | NA | NA | NA | 45 | 20 | 14 | 26 | 11 |
| Chen, Y. et al, 2022 [31] | 59 | NA | NA | NA | NA | 77 | NA | NA | NA | NA | 8 | NA | NA | NA | NA | 85 | NA | NA | NA | NA |
| Du, Y. et al, 2022 [28] | 647 | 37 | 28 | 65 | 17 | 493 | NA | NA | NA | NA | 102 | NA | NA | NA | NA | 595 | 291 | 190 | 324 | 87 |
| Guo, Y. et al, 2023 [19] | 518 | 165 | 128 | 305 | 127 | 596 | 263 | 213 | 400 | 219 | 148 | 83 | 74 | 102 | 104 | 744 | NA | NA | NA | NA |
| Lee, MJ. et al, 2013 [30] | 349 | 194 | 23 | 192 | 99 | NA | NA | NA | NA | NA | NA | NA | NA | NA | NA | 81 | 57 | 36 | 56 | 59 |
| Lim, CC. et al, 2020 [26] | 101 | 55 | 39 | 85 | 37 | 38 | 24 | 36 | 34 | NA | 6 | 6 | 6 | 6 | 6 | 44 | 30 | 42 | 40 | 24 |
| Ma, F. et al, 2020 [23] | 169 | 64 | 27 | 44 | 58 | 142 | NA | NA | NA | NA | 27 | NA | NA | NA | NA | 169 | 51 | 34 | 34 | 84 |
| Neves, PDMM. et al, 2020 [22] | 80 | 60 | 23 | 55 | 28 | 27 | NA | NA | NA | NA | 4 | NA | NA | NA | NA | 31 | 26 | 20 | 30 | 18 |
| Parka, S. et al, 2019 [14] | 2656 | NA | NA | 1435 | NA | 664 | NA | NA | 432 | NA | 60 | NA | NA | 25 | NA | 724 | NA | NA | NA | NA |
| Peng, W. et al, 2019 [13] | 993 | 747 | 12 | 473 | 234 | 257 | 220 | 25 | 155 | 68 | 78 | 76 | 27 | 47 | 26 | 335 | NA | NA | NA | NA |
| Ramani, N. et al, 2023 [20] | 47 | 26 | 5 | 34 | 15 | 21 | NA | NA | NA | NA | 5 | NA | NA | NA | NA | 26 | 25 | 8 | 17 | 8 |
| Ruan, Y. et al, 2022 [21] | 255 | 139 | 39 | 100 | 62 | 187 | 115 | 40 | 109 | 63 | 16 | 9 | 4 | 8 | 9 | 203 | NA | NA | NA | NA |
| Schimpf, JI. et al, 2018 [18] | 48 | NA | NA | NA | NA | 17 | NA | NA | NA | NA | 5 | NA | NA | NA | NA | 22 | NA | NA | NA | NA |
| Ştefan, G. et al, 2016 [25] | 83 | 55 | 16 | 53 | NA | NA | NA | NA | NA | NA | NA | NA | NA | NA | NA | 38 | 32 | 12 | 33 | NA |
| Wang, Z. et al, 2021 [27] | 16 | NA | NA | NA | NA | 24 | NA | NA | NA | NA | 60 | NA | NA | NA | NA | 84 | NA | NA | NA | NA |
| Zhang, W. et al, 2017 [29] | 554 | NA | NA | NA | NA | NA | NA | NA | NA | NA | NA | NA | NA | NA | NA | 434 | NA | NA | NA | NA |
| Zhang, X. et al, 2018 [12] | 619 | 208 | 157 | 422 | 195 | 447 | 217 | 239 | 363 | 133 | 86 | 44 | 63 | 54 | 36 | 533 | NA | NA | NA | NA |

**Supplementary Table 4. Network meta-analyses of renal outcomes among groups** (RR or MD, 95%CIs)

| **Composite kidney endpoint** | | |
| --- | --- | --- |
| **C0** | 1.15 (0.84,1.57) | 2.48 (1.75,3.53) |
| 0.87 (0.64,1.19) | **C1** | 2.17 (1.51,3.10) |
| 0.40 (0.28,0.57) | 0.46 (0.32,0.66) | **C2** |
| **ESRD** | | |
| **C0** | 1.00 (0.80,1.25) | 2.23 (1.74,2.85) |
| 1.00 (0.80,1.24) | **C1** | 2.23 (1.71,2.90) |
| 0.45 (0.35,0.57) | 0.45 (0.35,0.58) | **C2** |
| **eGFR** | | |
| **C0** | -3.14 (-8.36,2.07) | -20.36 (-26.67,-14.04) |
| 3.14 (-2.07,8.36) | **C1** | -17.21 (-23.62,-10.81) |
| 20.36 (14.04,26.67) | 17.21 (10.81,23.62) | **C2** |
| **Proteinuria** | | |
| **C0** | 0.29 (0.18,0.39) | 1.60 (1.36,1.84) |
| -0.29 (-0.39,-0.18) | **C1** | 1.31 (1.07,1.55) |
| -1.60 (-1.84,-1.36) | -1.31 (-1.55,-1.07) | **C2** |
| **M1** | | |
| **C0** | 1.25 (1.11,1.40) | 1.46 (1.28,1.67) |
| 0.80 (0.72,0.90) | **C1** | 1.17 (1.03,1.34) |
| 0.68 (0.60,0.78) | 0.85 (0.75,0.97) | **C2** |
| **E1** | | |
| **C0** | 2.31 (1.18,4.53) | 3.57 (1.80,7.11) |
| 0.43 (0.22,0.85) | **C1** | 1.55 (0.78,3.05) |
| 0.28 (0.14,0.56) | 0.65 (0.33,1.28) | **C2** |
| **S1** | | |
| **C0** | 1.20 (1.10,1.31) | 1.09 (0.97,1.22) |
| 0.83 (0.76,0.91) | **C1** | 0.90 (0.80,1.02) |
| 0.92 (0.82,1.03) | 1.11 (0.98,1.25) | **C2** |
| **T1/T2** | | |
| **C0** | 1.23 (0.96,1.59) | 2.01 (1.56,2.58) |
| 0.81 (0.63,1.05) | **C1** | 1.63 (1.24,2.14) |
| 0.50 (0.39,0.64) | 0.61 (0.47,0.81) | **C2** |

**Supplementary Figure 1. Meta-analyses of eGFR (A), proteinuria (B) and pathological characteristics (C) between C0 and C groups**

A.


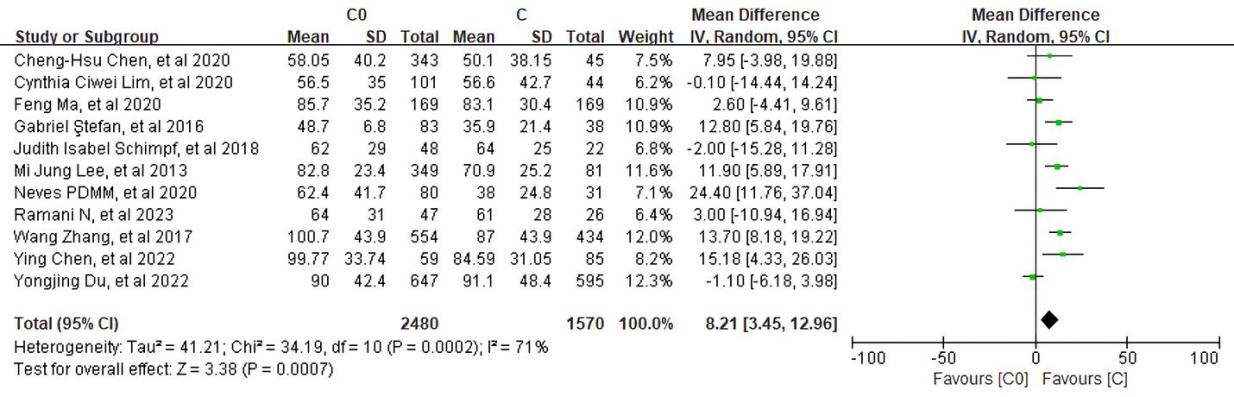


B.


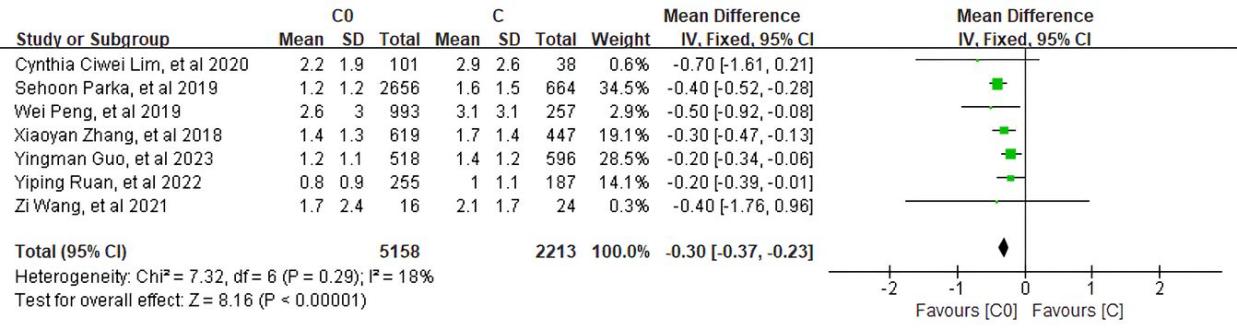


C.


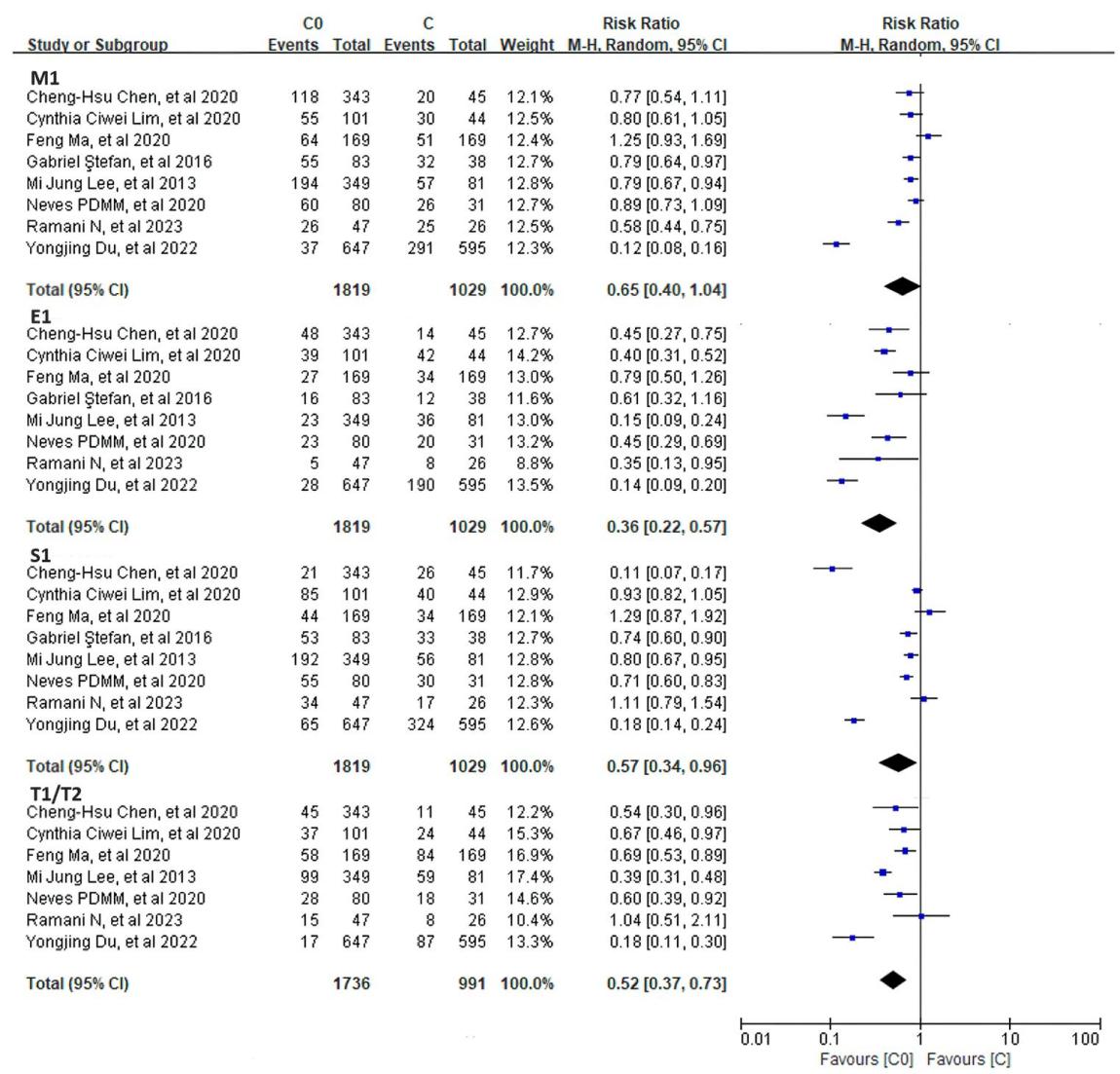

Supplement: Supplemental Material [file IRNF_A_2495104_SM9262.docx]
