# Supplementary figures and images for: Association between different proportions of crescents and adverse renal outcomes in immunoglobulin a nephropathy: a systematic review and meta-analysis
Source: Ren Fail. 2025 May 21;47(1):2495104. doi: 10.1080/0886022X.2025.2495104 (PMC12168408; doi:10.1080/0886022X.2025.2495104)

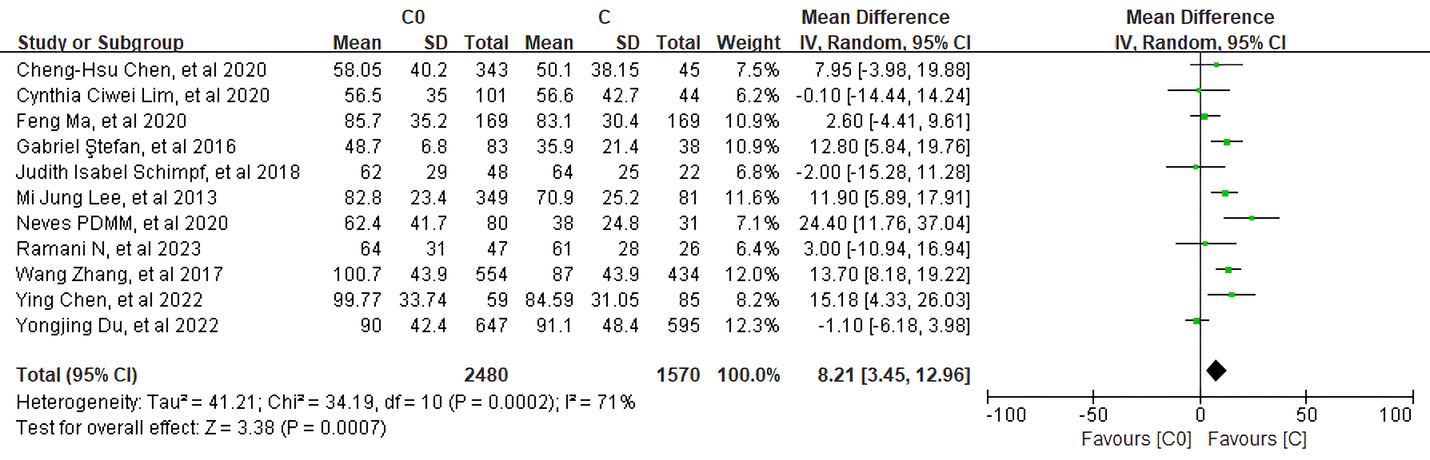

Supplement: Supplemental Material [file IRNF_A_2495104_SM7531.jpeg]

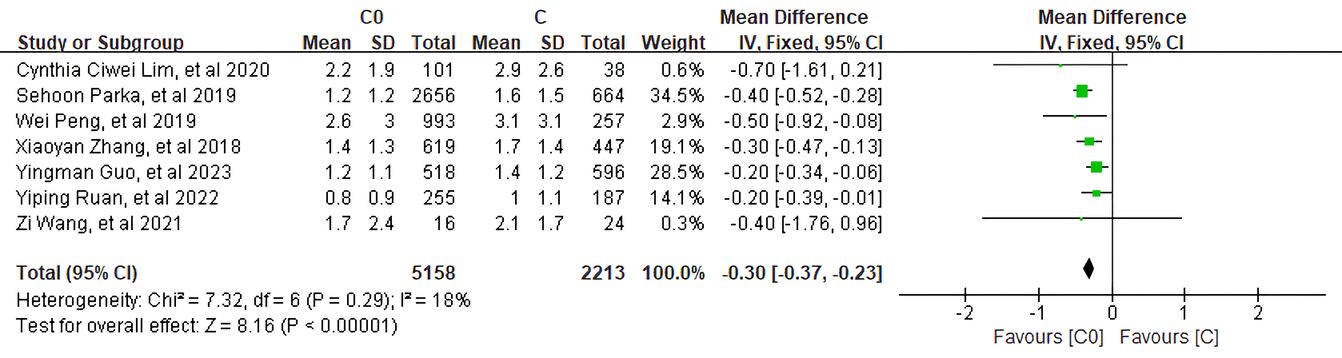

Supplement: Supplemental Material [file IRNF_A_2495104_SM7528.jpeg]

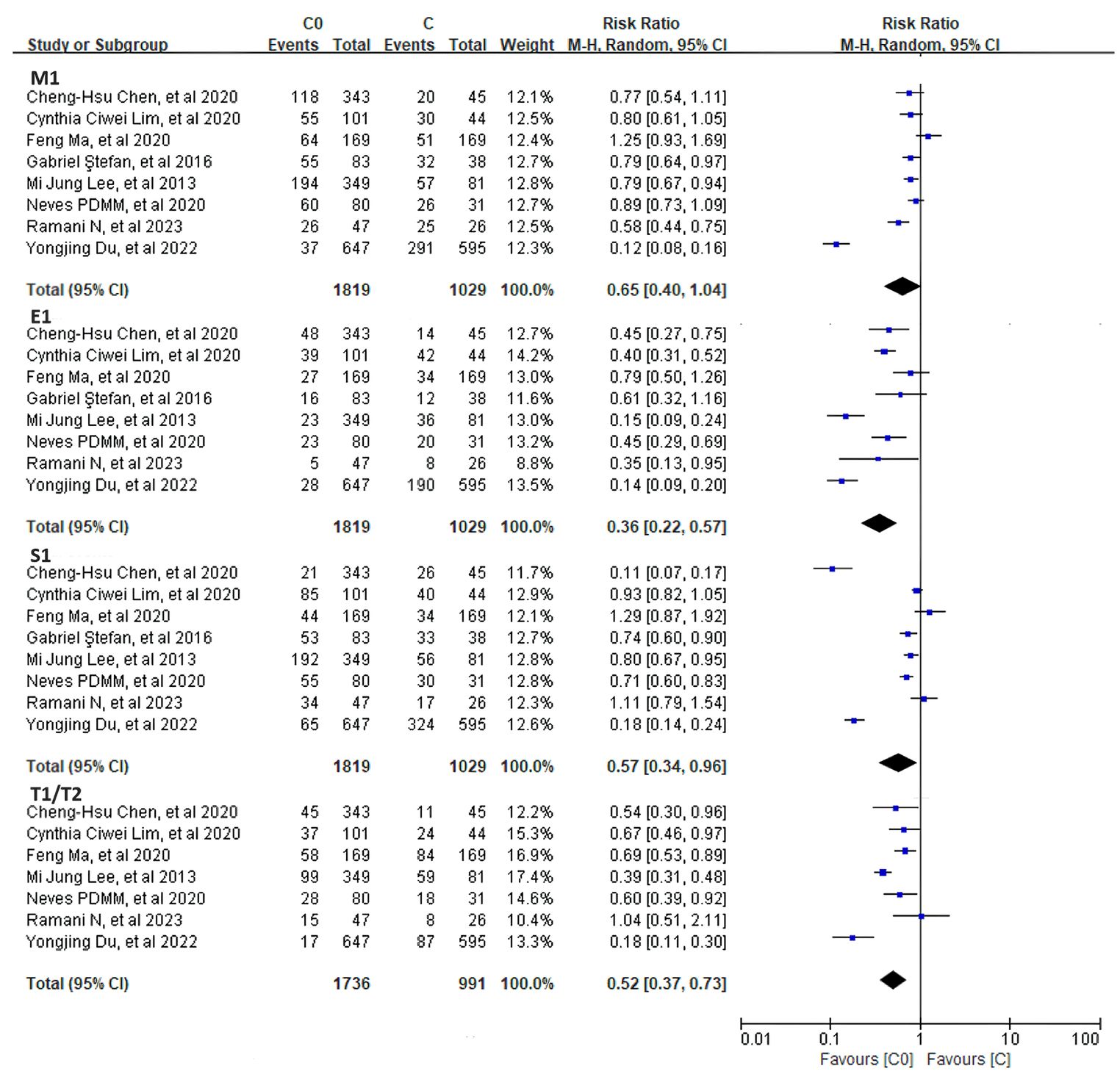

Supplement: Supplemental Material [file IRNF_A_2495104_SM7525.jpeg]
